# Deletion of Proton Gradient Regulation 5 (PGR5) and PGR5-Like 1 (PGRL1) proteins promote sustainable light-driven hydrogen production in *Chlamydomonas reinhardtii* due to increased PSII activity under sulfur deprivation

**DOI:** 10.3389/fpls.2015.00892

**Published:** 2015-10-27

**Authors:** Janina Steinbeck, Denitsa Nikolova, Robert Weingarten, Xenie Johnson, Pierre Richaud, Gilles Peltier, Marita Hermann, Leonardo Magneschi, Michael Hippler

**Affiliations:** ^1^Institute of Plant Biology and Biotechnology, University of MünsterMünster, Germany; ^2^Laboratoire de Bioénergétique et Biotechnologie des Bactéries et Microalgues, Institut de Biologie Environnementale et de Biotechnologie, Direction des Sciences du Vivant, Commissariat à l’Energie Atomique et aux Energies AlternativesSaint-Paul-lez-Durance, France; ^3^CNRS, UMR 7265, Biologie Végétale et Microbiologie EnvironnementaleSaint-Paul-lez-Durance, France; ^4^Laboratoire de Bioénergétique et Biotechnologie des Bactéries et Microalgues, Aix Marseille UniversitéSaint-Paul-lez-Durance, France

**Keywords:** PGR5, PGRL1, hydrogen production, PSII stability, sulfur deprivation

## Abstract

Continuous hydrogen photo-production under sulfur deprivation was studied in the *Chlamydomonas reinhardtii pgr5 pgrl1* double mutant and respective single mutants. Under medium light conditions, the *pgr5* exhibited the highest performance and produced about eight times more hydrogen than the wild type, making *pgr5* one of the most efficient hydrogen producer reported so far. The *pgr5 pgrl1* double mutant showed an increased hydrogen burst at the beginning of sulfur deprivation under high light conditions, but in this case the overall amount of hydrogen produced by *pgr5 pgrl1* as well as *pgr5* was diminished due to photo-inhibition and increased degradation of PSI. In contrast, the *pgrl1* was effective in hydrogen production in both high and low light. Blocking photosynthetic electron transfer by DCMU stopped hydrogen production almost completely in the mutant strains, indicating that the main pathway of electrons toward enhanced hydrogen production is via linear electron transport. Indeed, PSII remained more active and stable in the *pgr* mutant strains as compared to the wild type. Since transition to anaerobiosis was faster and could be maintained due to an increased oxygen consumption capacity, this likely preserves PSII from photo-oxidative damage in the *pgr* mutants. Hence, we conclude that increased hydrogen production under sulfur deprivation in the *pgr5* and *pgrl1* mutants is caused by an increased stability of PSII permitting sustainable light-driven hydrogen production in *Chlamydomonas reinhardtii*.

## Introduction

Solar fuels are an important motive for the development of future renewable energy systems with zero CO_2_ emission. This development is necessary to meet one of the most urgent challenges of our society today, to counter the problems of global warming, fossil fuel depletion, concurrent increasing energy demand, and consequently the maintenance of economic and political stability (Organisation for Economic Co-operation and Development (OECD)/International Energy Agency (IEA), 2011). Among other fuels, hydrogen is considered to be one of the most effective and clean fuels (Hankamer et al., 2007). Solar-driven H_2_ production by photosynthetic microorganisms, particularly cyanobacteria and microalgae, is a promising complement to clean and sustainable technologies of hydrogen production beside chemical techniques.

Photobiological hydrogen production was first discovered by Gaffron and Rubin, 1942. In this process, electrons and protons from water splitting are directed via photosynthesis toward specific H_2_-evolving enzymes, the hydrogenases. The algal Fe-Fe hydrogenase is very efficient compared to other hydrogenases (turnover rate in thousands per second, 100-fold higher than other hydrogenases; Volgusheva et al., 2013; Lubitz et al., 2014). However, direct light-to-hydrogen conversion efficiency is very low, because hydrogenase activity is extremely sensitive to oxygen (Ghirardi et al., 1997; Rupprecht et al., 2006; Stripp et al., 2009), thus oxygenic photosynthesis cannot easily be directly coupled to hydrogen production in green algae. Therefore, hydrogen production is a transient phenomenon in nature and stops after a few minutes of illumination (Bishop and Gaffron, 1963).

Melis et al. (2000) proposed an experimental protocol for prolong H_2_ evolution based on sulfur deprivation that circumvents this limitation. This method allows the separation of photosynthetic oxygen evolution and hydrogen production by a two stage process: In the first phase of cell cultivation, oxygenic photosynthesis drives production of biomass and carbohydrate stores in the presence of acetate as an additional carbon source. Shifting cells to sulfur depleted medium in sealed flasks induces the switch to the second anaerobic stage, inducing hydrogenase expression and sustainable H_2_ production for several days. During acclimation to this nutrient stress, cells stop dividing and undergo morphological changes (Zhang et al., 2002). Both light and dark reactions of photosynthesis are down-regulated, with the amounts of Rubisco being substantially reduced within the first 24 h of sulfur starvation (Zhang et al., 2002). Photosystem II activity drops gradually, attributed to an impaired PSII repair cycle due to the restricted *de novo* synthesis of the D1 reaction center protein (which contains methionines and cysteines) by S-limiting conditions, driving down O_2_ evolution (Wykoff et al., 1998). When O_2_ consumption overtakes O_2_ evolution, anaerobic conditions are established. However, active PSII in the first hours of S-depletion was shown to be essential for H_2_ generation, as no hydrogen evolution could be observed when the PSII inhibitor DCMU was added directly after transfer to S-free medium (Fouchard et al., 2005; Hemschemeier et al., 2008). Indeed, PSII activity was reported to contribute a substantial amount of electrons from water oxidation at PSII to hydrogen production (Antal et al., 2003; Kosourov et al., 2003). Beside this direct PSII-dependent pathway, electrons toward the hydrogenase can also derive from an indirect pathway that relies on non-photochemical reduction of PQs from metabolites such as starch (Fouchard et al., 2005; Jans et al., 2008; Chochois et al., 2009). Starch is massively accumulated during the first hours of –S conditions and is subsequently degraded (Zhang et al., 2002). The direct sunlight to hydrogen pathway has nevertheless the potential for higher energy conversion efficiency.

The improvement in hydrogen production by the method of sulfur deprivation implicated the highest efficiency for photobiological systems reported by then (Rupprecht et al., 2006). Yet, this H_2_ production efficiency has still to be advanced to reach economic profitability. Optimization of the electron supply to the hydrogenase appears to be a critical issue. Since the majority of electrons toward the hydrogenase have been shown to derive from PSII activity, but O_2_ evolution by PSII has to be prevented not to inhibit hydrogenase activity, possible solution scenarios involve either a more O_2_ tolerant hydrogenase or a more efficient oxygen scavenging system. Engineering resulting in a decrease in the O_2_ sensitivity of the Fe–Fe hydrogenase has not been reported yet. Among some other *Chlamydomonas reinhardtii* mutant strains, the *state transition 6* (*stm6*) mutant lacking the mitochondrial respiratory chain assembly factor *moc1*, and the *proton gradient regulation like 1* (*pgrl1*) mutant displayed the highest improved hydrogen production rates (Kruse et al., 2005; Tolleter et al., 2011). The PGRL1 protein was first discovered in *Arabidopsis* (DalCorso et al., 2008) as an essential component of the PGR5 (proton gradient regulation 5) dependent cyclic electron flow (CEF) pathway (Munekage et al., 2002). Moreover, PGRL1 has been suggested to operate as the elusive ferredoxin-plastoquinone reductase (Hertle et al., 2013). In *Chlamydomonas*, PGRL1 is also required for effective CEF (Petroutsos et al., 2009; Tolleter et al., 2011) and suggested to be a functional component of a CEF-supercomplex formed under CEF promoting conditions (Iwai et al., 2010; Terashima et al., 2012). A *pgr5* mutant has recently been characterized in *Chlamydomonas* (Johnson et al., 2014), which revealed that PGR5 deficiency leads to a diminished proton gradient across the thylakoid membrane accompanied with less effective CEF capacity. The proton gradient across the thylakoid membrane was shown to restrict electron flow toward the hydrogenase in *pgrl1*, and also proton uncouplers like carbonyl cyanide-*p*-trifluoromethoxyphenylhydrazone (FCCP) and nigericin increased hydrogen evolution (Antal et al., 2009; Tolleter et al., 2011). Interestingly, both *stm6* and *pgrl1* share the characteristic of an increased respiration rate (Kruse et al., 2005; Petroutsos et al., 2009; Dang et al., 2014). Enhanced H_2_ production in *stm6* was recently linked to prolonged PSII activity under sulfur starvation (Volgusheva et al., 2013). In this study, we investigated hydrogen production under similar conditions in the PGR5-deficient mutant and a *pgr5 pgrl1* double mutant in comparison to the *pgrl1* mutant. These mutants also exhibit enhanced hydrogen production and higher PSII stability under –S conditions. Since blocking PSII activity by DCMU abolished hydrogen production almost completely, we conclude that electrons for enhanced hydrogen production mainly derive from PSII. Anaerobiosis and therefore hydrogenase activity can still be maintained due to a higher oxygen consumption capacity in the mutants.

## Materials and Methods

### Strains and Growth Conditions

The *Chlamydomonas reinhardtii pgrl1* (Tolleter et al., 2011) mutant was back-crossed four times with the wild type (wt) strain CC124 (137c, nit2^-^, mt^-^) and then mated to *pgr5* (137c background; Johnson et al., 2014) in order to obtain *pgr5 pgrl1* double mutants in the 137c background. Zygotes were germinated on paromomycin plates (10 mg mL^-1^) and progeny was screened for both insertion of the *AphVIII* resistance marker within the *PGRL1* coding sequence (primers AphVIII_Fw1, 5′-TCGGGCCGGAGTGTTCC-3′ and PGRL1_Rev, 5′-TTACGCAGCGGCCTTAGCC-3′) and deletion of the PGR5 locus (primers PGR5 FW2, 5′-CTACTCGCAGCCAAAACACA-3′ and PGR5 REV2, 5′-GGAAACCAGTGTGCAAGTCA-3′). Mating type of the isolated clones was assessed with primers MID_Fw (5′-ATGGCCTGTTTCTTAGC-3′), MID_Rev (5′-CTACATGTGTTTCTTGACG-3′), FUS1_Fw (5′-ATGCCTATCTTTCTCATTCT-3′) and FUS1_Rev (5′-GCAAAATACACGTCTGGAAG-3′).

Wild-type, *pgrl1*, *pgr5*, and the *pgr5 pgrl1* double mutants were maintained on TAP medium (Harris, 1989), pH 7.0, solidified with 1.2% (w/v) agar at 25°C and 50 μE m^-2^ s^-1^ photosynthetically active, constant irradiance, and in case of the mutants supplemented with paromomycin (5 μg mL^-1^). Strains were cultured in standard TAP medium at 25°C under continuous light of 50 μmol photons m^-2^ s^-1^ on a rotary shaker (120 rpm).

### Long-term Hydrogen Production

Long-term hydrogen production was measured under conditions of sulfur deficiency (Melis et al., 2000). Cultures grown in TAP medium to a chlorophyll concentration of 20–25 mg L^-1^ were washed twice and resuspended (2500 × *g*, 5 min) in sulfur-deprived medium. Sealed in 250-ml glass flasks (Schott, conventional measuring system) or 1000-ml flasks (BlueSens gas measuring system) at a final chlorophyll concentration of 14–15 mg L^-1^, cells were incubated at room temperature, constant stirring and under continuous one-side illumination at medium light (60 μE m^-2^ s^-1^) or high light (200 μE m^-2^ s^-1^). If indicated, DCMU (10 μM final concentration) or Lincomycin (2 mM final conc.) were added 48 h after sealing. For the conventional measuring system, the gas phase was removed daily, with H_2_ and O_2_ concentrations analyzed from a 0.2 ml gas sample each day by gas chromatography (GC-2010, Shimadzu). Liquid samples (1 ml) were taken for immunoblot and pulse-amplitude modulated (PAM) chlorophyll fluorometry analysis. For the BlueSens gas measuring system, the gas phase was constantly removed and analyzed every 20 s with online gas sensors (BCP, BlueSens gas sensor GmbH).

### Chlorophyll Fluorescence Measurements

Fluorescence was measured at room temperature using a Maxi-Imaging PAM chlorophyll fluorometer (Heinz Walz GmbH). Samples were dark adapted for 20 min to maximally oxidize Q_A_ before each measurement. The effective photochemical quantum yield of PSII was measured as PSII yield [Y(II) = (F_m_′ – F)/F_m_′].

### Immunoblot Analysis

Whole cell samples (50 μg total protein, measured by Pierce^®^ BCA^TM^ Protein Assay Kit) were analyzed by discontinuous 13% SDS-PAGE according to Laemmli (1970) and transferred to nitrocellulose membrane (Hybond ECL membrane, GE Healthcare), which was incubated with antibodies against PSAD (1:1000, Naumann et al., 2005), ATPB CF1 (1:10 000, Agrisera) and PSBA D1 (1:2500, Agrisera) as described (Hippler et al., 2001; Naumann et al., 2005). Secondary antibody was anti-rabbit (Invitrogen) and signal detection was by enhanced chemical luminescence (ECL).

### Mass Inlet Membrane Spectroscopy (MIMS) Analysis of O_2_ Exchange

Mass Inlet Membrane Spectroscopy (MIMS) analysis was performed as described in Johnson et al. (2014) and a detailed description of the protocols for analyzing MIMS data are provided in Cournac et al. (2002). For O_2_ exchange experiments, cultures were grown in TAP at 10 μmol photons m^-2^ s^-1^ illumination. They were then centrifuged and resuspended in Minimal media and incubated for 24 h in 120 μmol photons m^-2^s^-1^ of light with 2% CO_2_ in an INFORS. Cells were then centrifuged and resuspended in fresh minimal medium without the addition of HCO_3_^-^ to a concentration of 10 mg mL^-1^ chlorophyll and left in the dark for 30 min before performing the experiments; 1.5 mL of the concentrated culture was added to the MIMS cuvette. Cells were incubated in the dark in the cuvette until the oxygen isotopes were in equilibrium and then exposed to a single light intensity at a time for around 10 min.

## Results

The depletion and/or absence of the PGRL1 induced increased hydrogen production under sulfur-deficiency in *Chlamydomonas reinhardtii* (Tolleter et al., 2011). Here, we investigate hydrogen production under similar conditions in a PGR5-deficient mutant and a *pgr5 pgrl1* double mutant.

### Generation of the *pgr5 pgrl1* Double Mutant

Both *pgr5* (Johnson et al., 2014) and *pgrl1* (Tolleter et al., 2011) were obtained by insertional mutagenesis with the *AphVIII* cassette, conferring resistance to the antibiotic paromomycin. In *pgr5*, integration of this cassette resulted in a complete deletion of the PGR5 locus, a phenomenon already observed in other insertional mutants (Peers et al., 2009; Johnson et al., 2014). In *pgrl1*, on the other hand, insertion of the *AphVIII* cassette occurred at the level of the first exon, resulting in lack of accumulation of the PGRL1 protein (Tolleter et al., 2011; Kukuczka et al., 2014). We took advantage of these genetic differences to screen for *pgr5 pgrl1* double mutants. Primers spanning the first 500 bp of the *PGR5* locus were used to differentiate wt-like (band) from *pgr5* (no band) progeny (Supplementary Figure S1). To select for absence of the PGRL1 protein in this *pgr5* background, we assessed integration of the resistance cassette inside the first exon of the *PGRL1* gene by *AphVIII*- and *PGRL1*-specific primers. Successful amplification of a ∼800 bp fragment represents the signature of the *pgrl1* mutant background (Supplementary Figure S1). From this screening, three independent *pgr5 pgrl1* double mutants were isolated, two in the mating type minus (mt–) and one in the mating type plus (mt+). The mt– *pgr5 pgrl1* double mutant #15 was further characterized at the physiological level, since it revealed the highest H_2_ production rates in a pre-screening test.

### Enhanced Long-term Hydrogen Production in the *pgr* Mutants under Different Light Conditions

**FIGURE 1:**
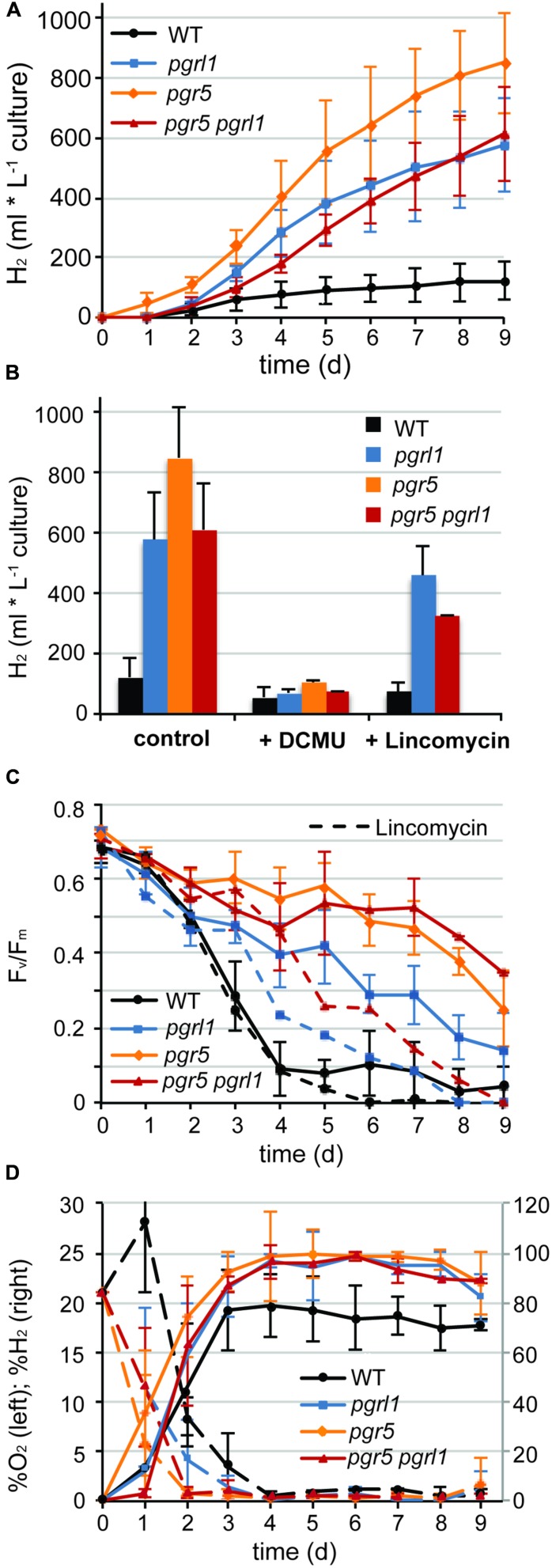
**Continues hydrogen production is enhanced in *pgr5*, *pgrl1*, *pgr5 pgrl1* compared to the wild type. (A)** Long-term hydrogen production of *pgr5*, *pgrl1*, *pgr5 pgrl1* and wild type under sulfur deprivation illuminated at 60 μmol photons m^-2^ s^-1^ (*n* = 4). **(B)** Total hydrogen volumes produced by *pgr* mutants and the wild type after 9 days of sulfur deprivation in the presence of DCMU (10 μM final conc.) or lincomycin (2 mM final conc.) compared to the control (*n* = 2). **(C)** Fv/Fm values measured after dark acclimation for 20 min of 200 μL aliquots from the S-deprived cultures (*n* = 4). **(D)** O_2_ (left axis) and H_2_ (right axis) concentrations of the gas phase of the cultures measured daily by gas chromatography. Concentrations remained constant after cells reached complete anaerobiosis on day 4 (*n* = 3). Data show mean ± SD.

**FIGURE 2:**
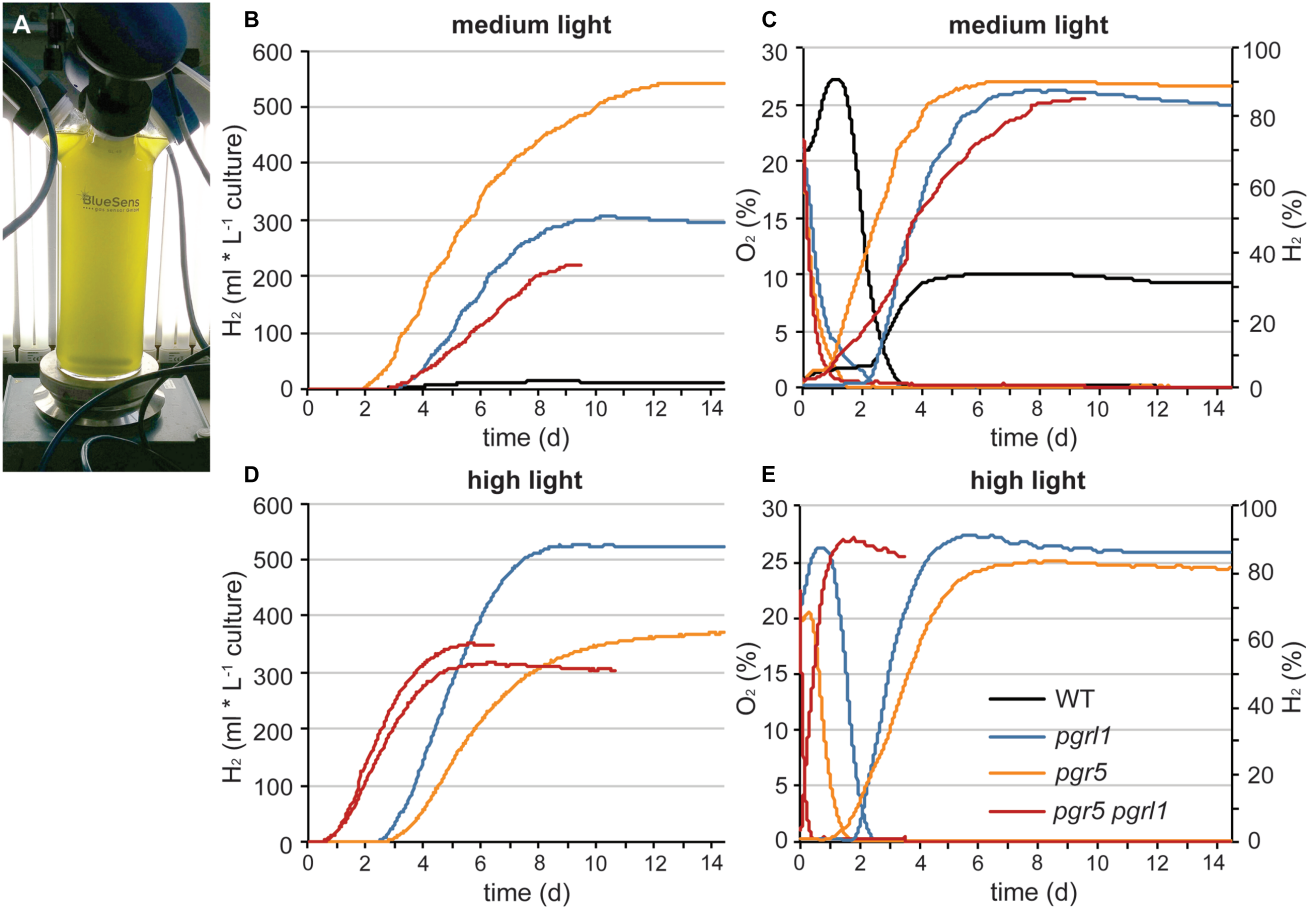
**Long-term hydrogen production under sulfur deprivation at different light conditions acquired with the continuous BluSens gas measuring system. (A)** Picture of the BlueSens 1 L-photobioreactor (PBR) gas measuring system. **(B,D)** represent continuous hydrogen production of *pgr5*, *pgrl1*, *pgr5 pgrl1* and the wild type at medium light (60 μmol photons m^-2^ s^-1^, *n* = 1) and high light (200 μmol photons m^-2^ s^-1^, *n* = 1, 2 for *pgr5 pgrl1*), respectively. **(C,E)** show the corresponding O_2_ (left axis, dotted line) and H_2_ (right axis, solid line) concentrations of the gas phase of the cultures measured every 20 s for medium and high light, respectively. Since gas concentrations remained constant after day 8, data are only shown till day 9 here (*n* = 1).

Continuous hydrogen production under sulfur deprivation was measured with two independent measuring systems over a period of up to 14 days (**Figures 1A** and **2**). The first approach used sealed flasks with a gas collection apparatus similar to that previously reported (Tolleter et al., 2011; **Figure 1**) from which gas was removed on a daily basis and analyzed using gas chromatography. Second, a continuous gas measuring system (BCP, BlueSens gas sensors GmbH) attached to the culture flasks was tested (**Figure 2**). Data collected with the BlueSens gas sensor system agreed well with data collected using the conventional apparatus, with a slight decrease in hydrogen production volumes (**Figures 1A** and **2B**). Continuous gas measurements have the ability to dissect more accurately the different phases of sulfur deprivation: (i) the O_2_ evolution phase, (ii) the O_2_ consumption phase, (iii) the anaerobic phase, and (iv) the H_2_ production phase. All four phases could be observed with the continuous BlueSens gas measuring system in the wild type, with a short transition from anaerobiosis to hydrogen production (**Figure 2C**), while the transition from oxygen consumption to complete anaerobiosis toward the onset of hydrogen production was less discrete using the conventional apparatus (**Figure 1D**). Both experimental setups, however, independently showed that oxygen consumption capacity was increased in the *pgr* mutants (**Figures 1D** and **2C**). Therefore, they reached anaerobiosis faster and started producing hydrogen earlier than the wild type, and stayed anaerobic during the remaining time of sulfur deprivation (**Figures 1A,D** and **2B,C**). This was especially pronounced in the *pgr5* mutant under medium light conditions.

The *pgr* mutants showed enhanced hydrogen production with four times higher maximal production rates than the wild type (7 ml L^-1^ culture per hour, **Figures 1A** and **2**). The *pgr5* mutant produced 7–10 times more hydrogen than the wild type in continuous medium light (**Figures 1A** and **2B**). With an overall volume of more than 800 ml hydrogen per liter (at 15 μg ml^-1^ chlorophyll, **Figure 1A**), *pgr5* exceeded the hydrogen production of the *pgrl1* mutant by about 1.5 times (**Figure 1A**). Total hydrogen volumes by *pgrl1* of 600 ml L^-1^ culture correspond to ∼1.6 μmol H_2_ μg^-1^ chlorophyll and were therefore in good agreement with previous published results (Tolleter et al., 2011). The *pgr5 pgrl1* double mutant was as effective in hydrogen production as the *pgrl1* mutant under medium light.

**FIGURE 3:**
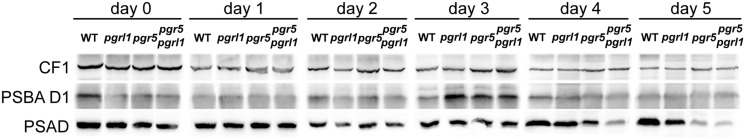
**Immunoblot analysis of the PSII-subunit PSBA D1 and the PSI-subunit PSAD during 5 days of sulfur deprivation in the wild type, *pgrl1*, *pgr5*, and *pgr5 pgrl1* mutant strains under high light conditions reveal that PSAD is severely degraded in *pgr5 pgrl1* and *pgr5*.** ATPase subunit CF1 served as loading control. Samples represent 50 μg total protein extract from high light exposed sulfur deprived cells. The corresponding hydrogen production and PSII maximum efficiency measurements are shown in Supplementary Figure S2.

Under elevated light conditions, oxygen evolution in *pgr5* and *pgrl1* was increased compared to medium light (**Figures 2C,E**), leading to a longer oxygen evolution phase. In contrast, the double mutant reached the anaerobic phase even faster (**Figure 2E**) and showed an increased hydrogen burst at the beginning of sulfur deprivation (**Figure 2D**). However, it stopped producing hydrogen much earlier compared to medium light conditions. Also *pgr5* produced less hydrogen when exposed to high light, while *pgrl1* remained effective in H_2_ production in both light settings (**Figure 2**, Supplementary Figure S2). Fractionation of whole cells by SDS-PAGE and immunoblotting using anti-PSAD and anti-PSBA antibodies revealed differences in the stability of the two photosystems (**Figure 3**). After 5 days of high light exposure under sulfur deprivation, the PSI complex in pgr5 was severely degraded as indicated by the strong decrease in the PSAD immunoblot signal (∼70%). In the absence of both PGR5 and PGRL1, this effect was even greater, since by day 4, PSI levels were already low and the double mutant stopped hydrogen production completely by day 5. At the same time, levels of PSII, which is known to be prone to degradation under sulfur deprivation (Melis et al., 2000), remained comparably stable up to day 5. Thus the observed decrease in hydrogen production in *pgr5* and the double mutant can be attributed to increased photosensitivity of PSI in the absence of PGR5. Moreover, the augmented decrease in PSI in the absence of PGR5 and PGRL1 indicate an additive effect between these two gene products, which are known to interact physically (DalCorso et al., 2008).

### The *pgr* Mutants show Prolonged PSII Activity and Increased Oxygen Consumption Capacity under Sulfur Deprivation Leading to Increased Hydrogen Production

**FIGURE 4:**
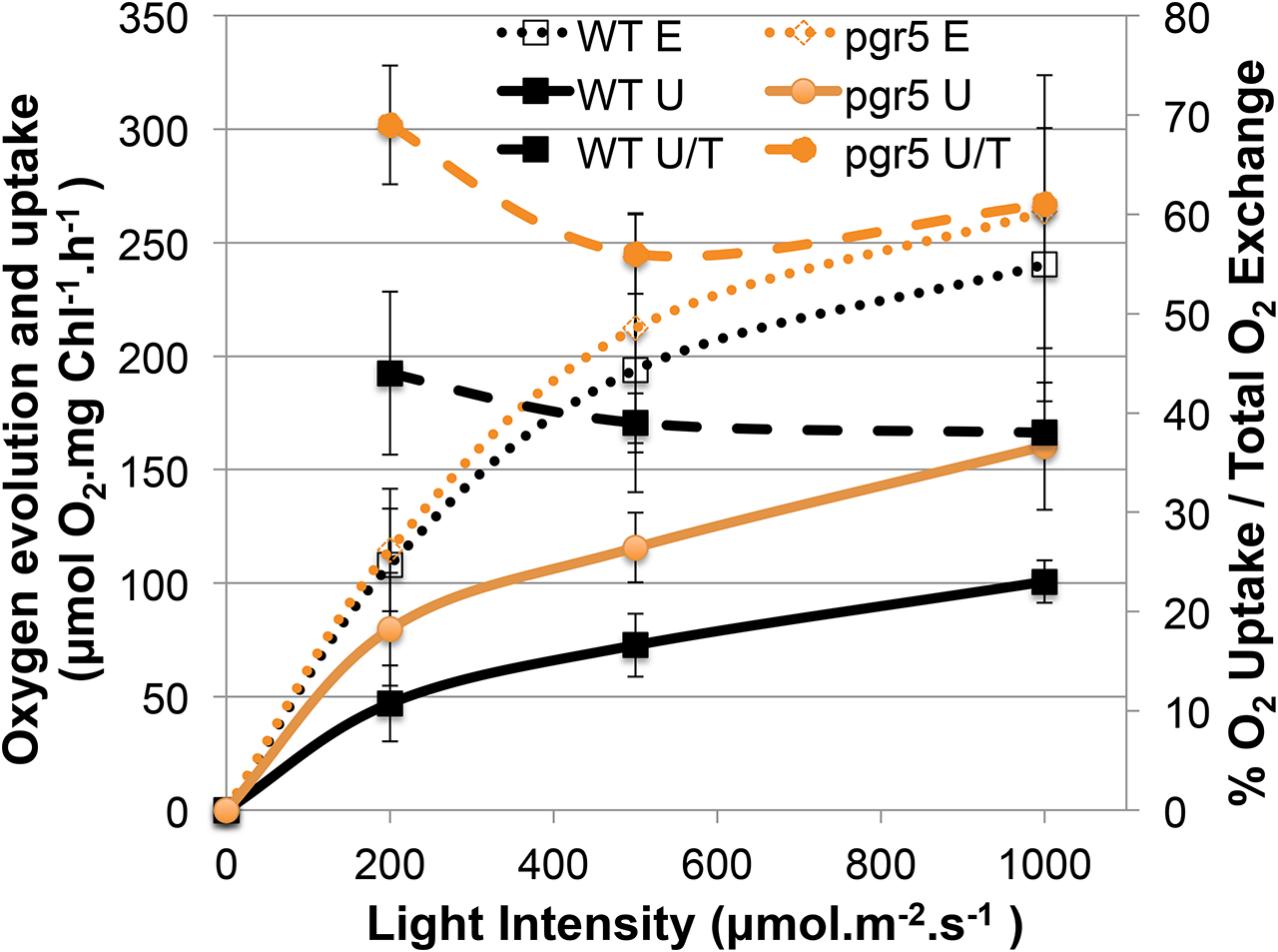
**Gas exchange analysis of competing O_2_ flows in the light comparing the wt and *pgr5* strains.** The left *y*-axis shows the rate of O_2_ Evolution (E) or O_2_ Uptake in the Light (U), normalized to the chlorophyll content of the cells. The right *y*-axis shows a ratio (U/T) between O_2_ Uptake in the light (U) and Total O_2_ exchange (U + E = T):. The *x*-axis shows the actinic light intensities used to excite photosynthesis in the cuvette. Data show mean ± SD (*n* = 3).

Sustainable hydrogen production under continuous light requires low oxygen concentrations due to the extremely oxygen sensitive Fe–Fe hydrogenases. Sulfur deprivation was reported to achieve anaerobiosis by down-regulating the activity of PSII (Melis et al., 2000). However, the maximum efficiency of PSII photochemistry, measured as the Fv/Fm values after 20 min of dark adaption (Baker, 2008), remained significantly higher in the *pgr* mutants under such sulfur limiting conditions compared to the control (wild type –S, **Figure 1C**). Despite this, *pgr* cells became anaerobic after 2 days of S deprivation, two times faster than the wild type (**Figures 1D** and **2D**). This suggests that in the *pgr* mutants, the driving force behind the more rapid attainment of anaerobiosis is not primarily the loss of PSII activity but instead is mediated by higher rates of respiration and light-dependent oxygen uptake, as demonstrated for *pgrl1* by Petroutsos et al. (2009), Tolleter et al. (2011), and Dang et al. (2014). This leads to a greater capacity to consume oxygen, even though some loss of PSII activity does occur. To test whether oxygen uptake is also increased in the *pgr5* mutant, we applied a MIMS technique that uses isotopic oxygen to differentiate between the rates of oxygen uptake in the light and photosynthetic oxygen evolution. Oxygen evolution (E) at different light intensities is similar between the wild type and *pgr5* showing that photosynthetic rates are not affected in *pgr5* under the conditions used (**Figure 4**, dotted lines). In contrast, oxygen uptake (U) rates in the light are higher at all light intensities in the *pgr5* strain compared to the wild type (**Figure 4**, solid lines). This is clearly demonstrated by the oxygen uptake in the light plotted as a percentage of total oxygen exchange (U/E + U = U/T): while wild type U/T is around 40%, the *pgr5* mutant is around 60%, with the highest tendency at the lowest light intensity. This supports the idea that respiratory and/or light induced oxygen photo-reduction pathways are considerably more active in the *pgr5* mutant than in the wild type.

The decline in Fv/Fm is similar between *pgr* mutants and wild type up to 1.5 days of sulfur deprivation. By the time the wild type reached complete anaerobiosis after 3.5 days of sulfur starvation, PSII activity already dropped to 10% (**Figures 1C,D** and **2D**). In contrast, PSII activity in the *pgr* mutants remained at ∼60–85% of the starting cultures by the time they reached anaerobiosis and remained higher compared to the wild type throughout the entire period of sulfur deprivation (**Figure 1C**). Thus, higher oxygen consumption capacities in the *pgr* mutants lead to faster induction and maintenance of anaerobiosis, thereby preserving PSII activity under –S conditions.

To test whether the electrons for increased hydrogen evolution could be derived from residual PSII activity in the *pgr* mutants, the inhibitor 3-(3,4-dichlorophenyl)-1,1-dimethylurea (DCMU) was added to the cultures after 2 days of sulfur starvation to block linear electron transfer at the level of the Q_B_ side of PSII. This time point was chosen to be able to distinguish between the contribution of the PSII-direct and indirect pathway of electrons toward the hydrogenase (Fouchard et al., 2005; Hemschemeier et al., 2008). The link between photosynthetic water oxidation and hydrogen production, and the mechanism behind it, is important for understanding photobiological hydrogen production and its further development (Volgusheva et al., 2013). Indeed, addition of DCMU blocked hydrogen production immediately (**Figure 1B**). Hydrogen volumes displayed in **Figure 1B** were mainly produced prior to addition of DCMU, with a residual amount of only 1% produced after adding DCMU. This indicates that electrons from linear photosynthetic electron transfer driven by the residual PSII activity contribute to hydrogenase-mediated H_2_-production, with elevated oxygen consumption being responsible for driving down O_2_ levels to achieve rapid anaerobiosis.

While the chlorophyll content remained stable in all strains, the chlorophyll *a/b* ratio of the wild type increased by 20% from 2.3 to 2.8 within 5 days of sulfur starvation. In contrast, the chlorophyll *a/b* ratio remained constant at 2.35 in all *pgr* mutants, an indication that PSII could be less degraded. In agreement, the PSBA amounts as revealed by the immunoblots analyses of sulfur-deprived cells showed a higher stability than the wt at certain time points in the *pgr* mutants (**Figure 3**). To further investigate whether PSII is more stable in the thylakoid membranes of the *pgr* mutants, the effect of the prokaryotic translation inhibitor lincomycin on PSII activity and hydrogen production was investigated (**Figures 1B,C**). Inhibiting chloroplast translation at day 2 of sulfur starvation reduced the PSII activity of the mutants as well as the wild type in a similar manner with 50% decreased Fv/Fm levels by day 5 compared to the sulfur deprived control samples (**Figure 1C**). The decrease in hydrogen production by 33% ± 10% was also similar between strains (**Figure 1B**). However, Fv/Fm levels and hydrogen production rates still remained higher in the *pgr* mutants compared to the wild type. This supports the conclusion that a substantial amount of electrons for hydrogen production in the *pgr* mutants derived from PSII. Moreover, as lincomycin had a similar effect on wild type as well as the *pgr* mutants, it can be assumed that the larger residual PSII activity under sulfur-deficiency is sustained due to the absence of PGR5 or PGRL1. However, it cannot be distinguished whether this is due to an altered repair cycle or a higher stability of PSII in the membrane.

## Discussion

In the present study, we investigated *pgr5* and *pgr5 pgrl1* double mutants in comparison to a PGRL1 deficient mutant and wild type in their capacity of light-driven hydrogen production. As already reported earlier for PGRL1 deficiency in *C. reinhardtii* (Tolleter et al., 2011), the absence of the *pgr5* gene product also increased the capacity of hydrogen production as compared to wild type significantly. Importantly, the *pgr5* mutant is, with hydrogen volumes of up to 10 times higher than the wild type, one of the highest hydrogen producers reported to date. The maximum amounts of 850 ml L^-1^ culture (at 15 μg ml^-1^ chlorophyll referring to 2.4 μmol H_2_ μg^-1^ chlorophyll) exceed reported volumes of *pgrl1* and other high hydrogen producers like *stm6*, *stm6glc4*, *LO1*, or the PSII D1 mutant *L159I-N230Y* (see **Table 1**, Kruse et al., 2005; Doebbe et al., 2007; Torzillo et al., 2009; Oey et al., 2013). Our data indicate that (i) the ability to maintain linear photosynthetic electron flow rates under S deplete conditions in conjunction with (ii) an increased oxygen consumption capacity explains the increased hydrogen production in the *pgr* mutants.

**Table 1:** Comparison of hydrogen amounts produced by different *Chlamydomonas* mutants.

Mutant	H_2_ volume (ml/L culture)	Chlorophyll (μg/ml)	H_2_ volume (μmol H2/μg chl)	PBR size (ml, ø in cm)	Reference
*pgr5*	850	15	2.4	250, 6.5	
*pgrl1*	580	15	1.6	250, 6.5	Also Tolleter et al., 2011
*pgr5 pgrl1*	610	15	1.7	250, 6.5	
*stm6*	540	26	0.9	500, 8.0	Kruse et al., 2005
*stm6 glc4*	150% of *stm6*	26	1.3	500, 8.0	Doebbe et al., 2007
*LO1*	400	15	1.1	500, 8.0	Oey et al., 2013
*L159I-N230Y*	504	12	1.75	1000, 5.0	Torzillo et al., 2009

*Absolute H_2_ volumes are taken from specified publications. For a better comparison, H_2_ amounts are equalized to chlorophyll content (column 4). Photobioreactor (PBR) sizes are given as one possible factor introducing some variance between studies due to differences in experimental set-ups.*

### Elevated Hydrogen Production in the pgr Mutants: Interplay between Capacities of Photosynthetic Electron Transfer and Oxygen Consumption

It is known that the hydrogenase is extremely sensitive to oxygen (Ghirardi et al., 1997; Stripp et al., 2009). On the other hand, linear electron transfer will produce oxygen. Compared to maximal wild type rates, the hydrogen amounts produced per hour were four times higher in the *pgr* mutants with maximal rates of 7 ml L^-1^ culture per hour over a period of 2–3 days (**Figure 1A**). The wild type stopped producing hydrogen slightly earlier than the mutants (**Figures 1A** and **2B**). After 2 days of sulfur deprivation (time point of DCMU addition, **Figure 1B**), the wild type produced already 46% vs. only 12% of hydrogen produced by the *pgr* mutants compared to the total hydrogen amounts produced over 9 days. This time point correlates with the drop in PSII activity in the wild type (**Figure 1C**). Thus, PSII activity of the *pgr* mutants remained higher until the late phase of sulfur deprivation (**Figures 1C** and **3**) suggesting that linear electron flow from PSII is responsible for the prolonged and significantly higher hydrogen production rates in the *pgr* mutants.

Why the hydrogenase is not inhibited by elevated PSII activity in the *pgr* mutants? This is because anaerobiosis can be maintained in the *pgr* mutants due to an increased oxygen consumption rate, as oxygen is removed much faster from the measuring system compared to wild type (**Figures 1D** and **2C,E**). This is further supported by the fact that light-induced oxygen uptake is increased in the *pgr5* mutant (**Figure 4**). Increased respiration rates were already reported for the *pgrl1 ko* and *kd* lines (Petroutsos et al., 2009; Tolleter et al., 2011). Recently, Dang et al. (2014) reported increased mitochondrial cooperation and increased O_2_ photo-reduction in the *pgrl1* mutant and also in *Arabidopsis pgr5*, this cooperation was enhanced (Yoshida et al., 2007). Metabolic shuttles such as the malate-oxaloacetate shuttle can export reducing power from the stroma to the mitochondria (Scheibe, 2004; Shen et al., 2006). This cooperation is particularly enhanced under stress conditions, where ATP demand is increased (Lemaire et al., 1988). Mitochondrial inhibitors induced a drop in the PSII yield in *pgrl1* (Dang et al., 2014). Also light-dependent O_2_ uptake and the abundance of flavodiiron proteins were higher in the mutant, indicating an increased capacity of Mehler-like reactions and possibly photorespiration. High antioxidant capacity was reported for *Arabidopsis pgr5* (Suorsa et al., 2012). A recent study showed an up-regulation of those proteins in the early acclimation phase of sulfur deprivation, suggesting an involvement of flavodiiron proteins in acclimation to anoxia during hydrogen production (Jokel et al., 2015). In addition to the very similar phenotype of the *pgrl1* and *pgr5* mutants in regard to CEF impairment, NPQ reduction and PSI photo-inhibition (Tolleter et al., 2011; Johnson et al., 2014; Kukuczka et al., 2014), O_2_ consuming mechanisms are also up-regulated under S-deplete mixotrophic conditions in *pgr5* (**Figures 1D**, **2D,E** and **4**) and participate in a faster anaerobic induction at the onset of sulfur deprivation (**Figures 1D** and **2D,E**). This is in line with the conclusion, that increased oxygen consumption rates allow higher PSII activity under anaerobic –S conditions leading to increased hydrogen production in the *pgr* mutants. Interestingly, in a recent study using the *stm6* and PSII-D1 *L159I-N230Y* mutant (see also **Table 1**), the authors reported very similar phenotypes under sulfur deprivation as is shown here for the *pgr* mutants: the mutants reached anaerobiosis faster and increased hydrogen production was mainly achieved by an increased electron supply from PSII toward the hydrogenase due to an increased respiration rate (Torzillo et al., 2009; Scoma et al., 2012; Volgusheva et al., 2013).

The contribution of the indirect pathway of electrons from starch via NAD2 toward the hydrogenase to H_2_ production has been reported to be variable, depending on experimental conditions and particularly on the phase of the sulfur deprivation process (Kosourov et al., 2003; Fouchard et al., 2005; Hemschemeier et al., 2008; Jans et al., 2008). It might also contribute to increased hydrogen production in the *pgr* mutants. Starch breakdown was reported to be faster in the *pgrl1* mutant (Tolleter et al., 2011) and from a study with a starch deficient mutant and the inhibitor DCMU and proton uncoupler FCCP, it was concluded that the proton gradient generated by cyclic electron flow around PSI inhibits the indirect pathway of electrons from starch to the hydrogenase (Chochois et al., 2009). Tolleter et al. (2011) reported hydrogen production rates in *pgrl1* are enhanced compared to the wild type, and to a similar extent independent of the presence of DCMU. However, H_2_ production was 10 times lower than in the absence of DCMU (Tolleter et al., 2011). In our hands, the hydrogen production in the presence of DCMU was even lower (**Figure 1B**), possibly because DCMU was added with a 1-day delay (48 h vs. 24 h of sulfur deprivation). Therefore, since blocking the PSII-dependent direct pathway by DCMU had such a dramatic effect on hydrogen production, the contribution of the indirect pathway to hydrogen production in the *pgr* mutants should be relatively small compared to the PSII-dependent pathway.

### Deletion of PGRL1 and PGR5 Sustains PSII Activity under –S Conditions

The mechanisms contributing to the inhibition of photosynthetic O_2_ evolution in S depleted conditions and their relative importance are still a matter of debate (Ghysels and Franck, 2010). Sulfur deprivation has a general effect on the transcription of the chloroplast (Irihimovitch and Stern, 2006; Irihimovitch and Yehudai-Resheff, 2008). It is well described that acclimation to sulfur deficiency is highly controlled and induces a down-regulation of photosynthesis, particularly of PSII (Davies et al., 1996). The absence of down-regulation of photosynthetic activity, as observed in the *sac1* mutant (Davies et al., 1996), is deleterious. It has been suggested that sulfur limitation will result in a general decrease in protein synthesis, which in turn would impact the capacity of D1 turn-over and repair cycle (Wykoff et al., 1998; Zhang et al., 2002). Hence, under low sulfur and in the absence of efficient PSII-repair, high activity of photosynthetic electron transfer would likely result in damage due to the production of reactive oxygen species, which could not be sustained by D1-repair. In the *pgr* mutants, however, PSII photochemistry efficiency remained high (**Figure 1C**) and amounts of the D1 protein in the mutants showed some stability compared to the wild type during sulfur starvation (**Figure 3**), at which PSII activity in the wild type had already dropped to 10% (**Figure 1C**). The inhibitory effect of chloroplastida translation by lincomycin on PSII activity and H_2_ production further indicates that a substantial amount of PSII remains functional under sulfur deprivation (**Figures 1B,C**). Thus, the absence of PGRL1 and PGR5 sustained PSII activity under sulfur deprivation. How could this be explained?

The increased oxygen consumption capacity in the mutants, and the faster transition to anaerobiosis is, in its own right, sufficient to explain PSII preservation, via at least two mechanisms. First, rapid attainment of anaerobiosis leads to early induction and high activity of the hydrogenase, the final electron acceptor of linear electron flow in –S conditions. This is important because CO_2_ fixation is lost during S deprivation, which normally limits LEF by slowing down the reoxidation of its main final electron acceptor NADP^+^: Rubisco is affected even earlier than D1 and declines by about 80% in the first 24 h of S deprivation, becoming undetectable after 60 h of starvation (Zhang et al., 2002). Loss of CO_2_-fixation has been reported to slow down the D1 repair cycle and was therefore predicted to stimulate the loss of PSII activity during S deprivation (Takahashi and Murata, 2005, 2006). In summary, early anaerobiosis releases acceptor side limitation of LEF faster due to an earlier activation of the hydrogenase (**Figures 1D** and **2D**). Second, faster induction of anaerobiosis at the onset of sulfur starvation and induction of hydrogenase activity preserves existing PSII from photo-oxidative damage, as it reduces the amount of oxygen available to cause potential damage at PSII. All strains show an initial decline in Fv/Fm, which slows after the system becomes anaerobic, but as this occurs much later in the wt, the corresponding damage to PSII is much worse.

### Under High Light Exposure, *pgr5 pgrl1* shows an Increased Hydrogen Burst at the Beginning of Sulfur Depletion and PSI becomes Photo-inhibited in the *pgr5* and *pgr5 pgrl1* Mutants

In this study, we report the generation of a *pgr5 pgrl1* double mutant. This double mutant displays a similar phenotype compared to its respective single mutants, confirming that PGR5 and PGRL1 both act on the same pathway as earlier proposed (DalCorso et al., 2008; Johnson et al., 2014). However, *pgrl1* and *pgr5* differ in the severity of one particular phenotype: While PSII remained stable in sulfur limiting conditions, PSI was degraded much faster in the *pgr5* and *pgr5 pgrl1* mutants when they were exposed to additional stress of high light (**Figure 3**), reducing the amount of hydrogen produced (**Figure 2**; Supplementary Figure S2). In contrast, *pgrl1* remained effective in hydrogen production also in high light and PSI was not degraded at the same rate (**Figures 2** and **3**). Although PGR5 was previously not detectable by western blot analysis in the *pgrl1* mutant (Johnson et al., 2014), the different phenotype of the *pgr5 pgrl1* and *pgr5* mutant compared to the *pgrl1* single mutant suggests that the PGR5 protein is present in the *pgrl1* mutant, though below the limit of detection of immunoblot analysis, which could detect about 50% of PGR5 wild type protein amounts (Johnson et al., 2014). High light sensitivity has already been reported for *pgr5* under photoautotrophic conditions (Johnson et al., 2014). In *pgrl1*, PSI becomes photo-inhibited under high light exposure (Kukuczka et al., 2014), although not degraded as severely as in *pgr5*. In the absence of both PGR5 and PGRL1, PSI becomes even more affected, indicating an additive role of these two proteins in terms of PSI protection and suggesting that both proteins operate in the same pathway.

PSI photo-inhibition in high light was also described in a *Chlamydomonas stt7-1* mutant locked in state 1 (Bergner et al., 2015). However, this phenotype was less severe than in the *pgr5* and *pgrl1* mutants (Johnson et al., 2014; Bergner et al., 2015). Furthermore, the *stt7-1* mutant displayed high CEF rates and enhanced formation of a CEF-supercomplex (Bergner et al., 2015). A non-successful acclimation to high light was recently also linked to altered dephosphorylation patterns of LHCII proteins in *Arabidopsis pgr5* (Mekala et al., 2015). These findings indicate a regulatory link between the PGR5/PGRL1 pathway, phosphorylation and dephosphorylation dynamics and the susceptibility of PSI to photo-inhibition.

In high light, the thylakoid lumen becomes acidified, which down-regulates linear electron flow and induces dissipation of excess energy at PSII in the form of heat. This so called non-photochemical quenching (NPQ) requires the induction of the LHCSR3 protein in algae (Peers et al., 2009) and acidification of the thylakoid lumen (Petroutsos et al., 2011). Both *pgr5* and *pgrl1* have reduced levels of NPQ due to a reduced proton gradient across the membrane, while LHCSR3 levels are unaltered in the mutants (Tolleter et al., 2011; Johnson et al., 2014). Without a sufficient proton gradient, linear electron flow is not down-regulated and the acceptor side of PSI becomes over-reduced. An indication that an increased electron drive toward the hydrogenase occurs under elevated light conditions is the enhanced hydrogen burst at the beginning of the hydrogen production phase in the *pgr5 pgrl1* mutant (**Figure 2C**). Hydrogen production at PSI was proposed to act as a safety valve to protect the photosynthetic electron transport chain from over-reduction under natural conditions by safely disposing of excess electrons from PSI (Melis and Happe, 2001; Happe et al., 2002; Hemschemeier et al., 2009). Now, the deletion of PGR5 and/or PGRL1 is deleterious for PSI under high light conditions. Thus, while PSII stability under sulfur deficiency is more affected in wild type, PSI integrity is most affected in the absence of PGR5 and/or PGRL1; a topic that certainly requires additional work for a more in-depth understanding.

## Conclusion

Enhanced hydrogen production rates in the *pgrl1, pgr5*, and the *pgr5 pgrl1* double mutant under S deplete conditions lead to the highest continuous photobiological produced hydrogen amounts of eukaryotic cells reported so far. These rates are achieved by a prolonged residual PSII activity providing an increased electron supply toward the hydrogenase. PSII activity can be maintained in the mutants without inhibiting the oxygen sensitive hydrogenase, because the oxygen consumption capacity is increased. Our results suggest that respiration and light-dependent O_2_-uptake rates are higher in the *pgr* mutants, and that this is responsible for the faster transition to anaerobiosis, especially when the greater residual PSII activity of the mutants is taken into account.

## Conflict of Interest Statement

The authors declare that the research was conducted in the absence of any commercial or financial relationships that could be construed as a potential conflict of interest.
