# Supplementary material for: Deletion of Proton Gradient Regulation 5 (PGR5) and PGR5-Like 1 (PGRL1) proteins promote sustainable light-driven hydrogen production in Chlamydomonas reinhardtii due to increased PSII activity under sulfur deprivation
Source: Front Plant Sci. 2015 Oct 27;6:892. doi: 10.3389/fpls.2015.00892 (PMC4621405; doi:10.3389/fpls.2015.00892)
Supplement: Supplementary file 1 [file Data_Sheet_1.DOCX]

Supplementary Material

**Deletion of Proton Gradient Regulation 5 (PGR5) and PGR5-Like 1 (PGRL1) proteins promote sustainable light-driven hydrogen production in *Chlamydomonas reinhardtii* due to increased PSII stability under sulfur deprivation**

Janina Steinbeck, Denitsa Nikolova, Robert Weingarten, Xenie Johnson, Pierre Richaud, Gilles Peltier, Marita Hermann, Leonardo Magneschi and Michael Hippler^*^

*** Correspondence:** Michael Hippler, mhippler@uni-muenster.de

# Supplementary Figures and Tables

## Supplementary Figures


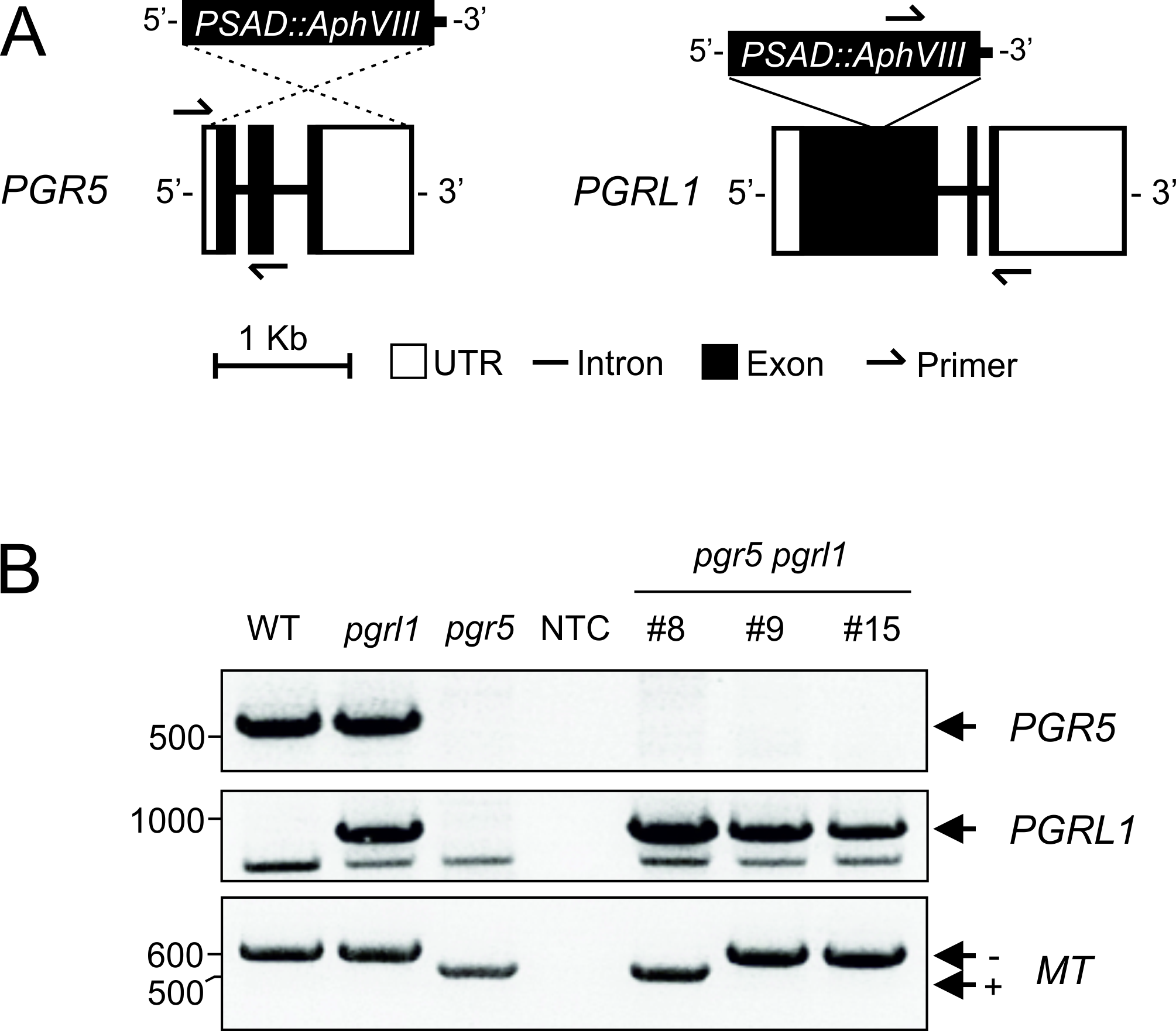


**Supplementary Figure 1. Generation of the Chlamydomonas *pgr5 pgrl1* double mutants. (A)** Schematic representation of the *PGR5* and *PGRL1* locuses and site of integration of the paromomycin resistance cassette in the respective mutants. Primers binding sites are indicated by arrows. **(B)** PCR-based screening on the obtained *pgr5 pgrl1* double mutants; parental *pgr5* and *pgrl1* single mutants and the CC124 wild type (WT) were also included. The presence of a 500 bp fragment in the top gel indicates presence of the *PGR5* locus, which is deleted in the *pgr5* mutant; the ~ 800 bp fragment shown in the middle gel arise from integration of the *AphVIII* cassette inside the *PGRL1* locus in the *pgrl1* mutant. Mating type of the different strains was also assessed (MT -, ~ 600bp; MT +, ~ 500bp). NTC, Non Template Control.

**
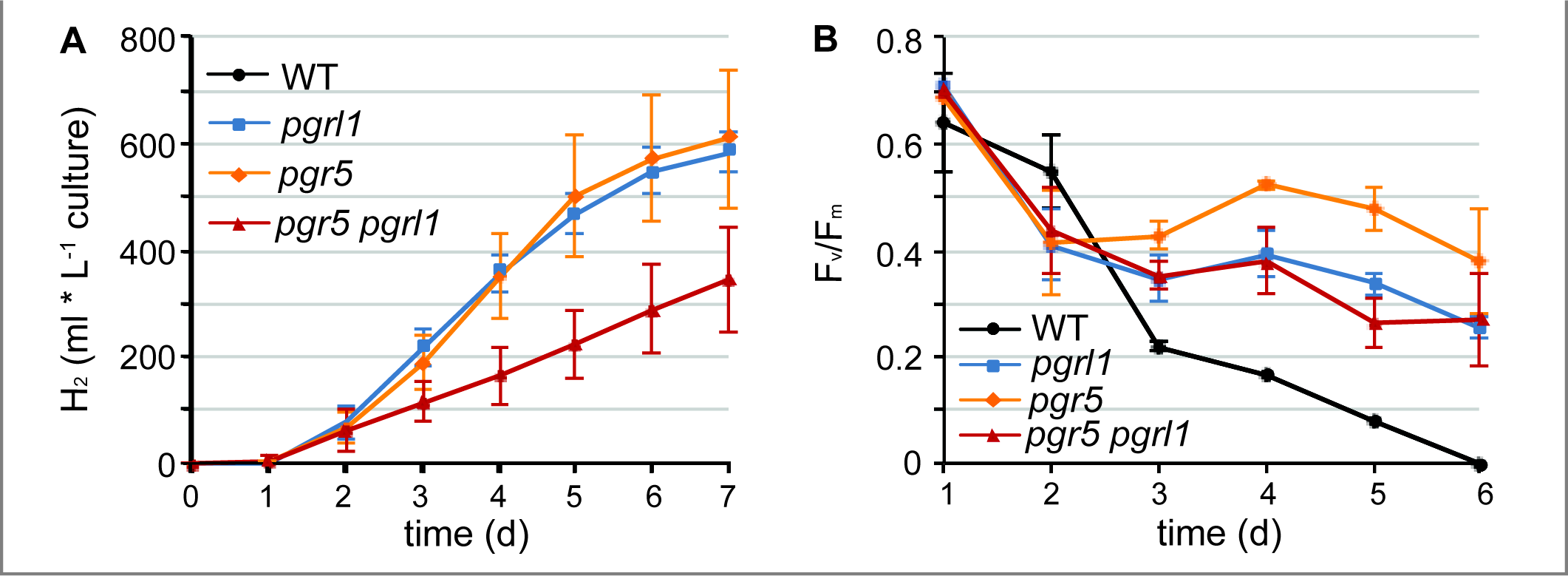
**

**Supplementary Figure 2. Long term hydrogen production of *pgr5*, *pgrl1*, *pgr5 pgrl1* compared to the wild type under high light conditions. (A)** Continuous hydrogen production under sulfur deprivation at 200 µE m^-2^ s^-1^ measured with the conventional measuring system as described in Materials and Methods (n = 3). **(B)** Fv/Fm values measured after dark acclimation for 20 min. of 200 µL aliquots from the S-deprived cultures (n = 3).
